# Impact of patrilocality on contrasting patterns of paternal and maternal heritage in Central-West Africa

**DOI:** 10.1038/s41598-024-65428-z

**Published:** 2024-07-08

**Authors:** Masinda Nguidi, Verónica Gomes, Carlos Vullo, Pedro Rodrigues, Martina Rotondo, Micaela Longaray, Laura Catelli, Beatriz Martínez, Afonso Campos, Elizeu Carvalho, Victoria O. Orovboni, Samuel O. Keshinro, Filipa Simão, Leonor Gusmão

**Affiliations:** 1 DNA Diagnostic Laboratory (LDD), State University of Rio de Janeiro (UERJ), Rio de Janeiro, Brazil; 2 Instituto de Investigação e Inovação em Saúde (i3S), Universidade do Porto, Porto, Portugal; 3 Institute of Pathology and Molecular Immunology, University of Porto (IPATIMUP), Porto, Portugal; 4 DNA Forensic Laboratory, Equipo Argentino de Antropología Forense (EAAF), Córdoba, Argentina; 5 Institute for Immunological Research, University of Cartagena, Cartagena, Colombia; 6 Department of Immunology and Inflammation, Imperial College London, London, UK; 7 Force Pathologist Office, Nigeria Police Force, Lagos, Nigeria; 8 Department of Forensic Science, Virginia Commonwealth University, Richmond, VA USA

**Keywords:** Genetics, Genetic markers, Haplotypes, Population genetics

## Abstract

Despite their ancient past and high diversity, African populations are the least represented in human population genetic studies. In this study, uniparental markers (mtDNA and Y chromosome) were used to investigate the impact of sociocultural factors on the genetic diversity and inter-ethnolinguistic gene flow in the three major Nigerian groups: Hausa (n = 89), Yoruba (n = 135) and Igbo (n = 134). The results show a distinct history from the maternal and paternal perspectives. The three Nigerian groups present a similar substrate for mtDNA, but not for the Y chromosome. The two Niger–Congo groups, Yoruba and Igbo, are paternally genetically correlated with populations from the same ethnolinguistic affiliation. Meanwhile, the Hausa is paternally closer to other Afro-Asiatic populations and presented a high diversity of lineages from across Africa. When expanding the analyses to other African populations, it is observed that language did not act as a major barrier to female-mediated gene flow and that the differentiation of paternal lineages is better correlated with linguistic than geographic distances. The results obtained demonstrate the impact of patrilocality, a common and well-established practice in populations from Central-West Africa, in the preservation of the patrilineage gene pool and in the affirmation of identity between groups.

## Introduction

As the homeland of the anatomically modern human, Africa harbors a high cultural, linguistic, and ethnic diversity^1^. This continent comprises almost a third of the world’s ethnolinguistic groups, with four major linguistic families distributed in continental Africa: Niger-Congo, Afro-Asiatic, Nilo-Saharan and Khoisan. While Niger-Congo groups are widespread in the African territory, the Afro-Asiatic, Nilo-Saharan and Khoisan families have a more restricted geographic distribution, being confined to northern/eastern, central/east, and south regions, respectively^1^. Studies on the genetic variation in African populations have been crucial for understanding the history of the continent, by clarifying hypotheses raised by historical, anthropological, and linguistic data. Nonetheless, given its ethnolinguistic variability the African continent can be considered understudied.

Nigeria, located in western Africa, in the Gulf of Guinea region (Supplementary Fig. S1), stands as a territory where multiple pivotal historical events have unfolded, contributing to the development of a population that upholds a diverse linguistic, archaeological, and genetic heritage^2^. The territory between Nigeria and Cameroon is thought to be the homeland of Bantu natives, which spread further through a vast region of the continent, leading to the spread of agriculture and metallurgy across sub-Saharan Africa^3,4^. Later, Nigeria was the region where the Nok people emerged, the most ancient civilization of sub-Saharan Africa (400 BCE until 200 CE)^2^.

Nigeria is currently the most populous African country and comprises a multiplicity of ethnolinguistic groups (at least 250 distinct groups currently exist in the country). The existence of the major current ethnolinguistic groups and their kingdoms in the country is dated before the fifteenth century. Due to their divergent beliefs and varied cultures, different dynamics and political organizations were implemented, which led to the geographic separation of the groups as they gradually emerged. The largest and most representative ethnolinguistic groups are the Hausa (representing 29% of the population), the Yoruba (20%) and the Igbo (17%)^5^.

The Hausa people belong to the Afro-Asiatic linguistic family and live mainly in the North of Nigeria (Supplementary Fig. S1), in the savannas where the ancient Hausaland kingdom was established (9th–tenth century CE). This ethnic group is an important member of the Chadic branch, and it is settled close to the Lake Chad Basin, a region that comprehends diverse ethnolinguistic populations. Their location also allowed contact with people from the Middle East and North Africa, during the Trans-Saharan trade, who introduced Islam to the Hausa people as result of cultural exchanges^2^.

The Yoruba and Igbo of the Niger-Congo linguistic family emerged in South Nigeria, in the former Yorubaland (Supplementary Fig. S1), and still inhabit this region. These groups had strong contact with Christian religious beliefs after the European arrival in the fifteenth century^2^.

Culturally, these three ethnic groups have patrilineal descent systems and practice polygyny, which is common to most traditional Nigerian societies^6^. Data retrieved from the Area Database of the Global Data Lab (https://globaldatalab.org/areadata, version v4.2.^7^) shows that patrilocality still prevails in Nigeria, with positive patrilocality indexes [log (% patrilocal/% matrilocal)] obtained in recent demographic surveys in all states.

The European colonization and the subsequent exploitation of the people and the Nigerian territory prompted interactions between different ethnic groups. During this period, while some groups in Nigeria were extinguished, others as the Hausa, Igbo, and Yoruba grew with the incorporation of people from more vulnerable groups^3^. Furthermore, with the European colonization, new political divisions in the country were implemented, which resulted in the current state limits.

The wide diversity of ethnicity, linguistic affiliation, and religious beliefs of the Nigerian groups had important political and social impacts. Several conflicts emerged over the years, due to religious and ethnic divergences. The Civil War (or Biafran War, 1967–1970) was the most important and brutal conflict in Nigeria that ended up with more than one million civilian deaths. The war started shortly after the Independence of the country (1960), because of inter-ethnic and inter-religious turmoil episodes, involving, mostly, Igbo and Hausa groups^2^.

The territory pluralistic/mosaic history has raised questions related to the impact of such historic events on the genetic differentiation of the current Nigerian population groups.

Genetic markers located on mitochondrial DNA (mtDNA) and non-recombining region of the Y chromosome (Y-Chr) allow the identification of maternal and paternal lineages, respectively, providing independent evolutionary histories. Thus, these markers are commonly used to provide information on population demographic history, sex-specific migrations, and mating patterns^8–12^. By combining information on mtDNA control region, Y-STRs and Y-SNPs, the main goal of this study was to investigate the impact of sociocultural factors such as matrimonial practices, cultural exchanges, inter-ethnolinguistic migrations, and post-colonial inter-ethnolinguistic conflicts on the genetic composition of Hausa, Yoruba and Igbo ethnic groups from Nigeria. These inquiries were further extended to encompass the Central-West African context, by supplementing our findings with data previously reported for other ethnolinguistic groups from Nigeria and neighboring countries.

## Material and methods

### Samples, DNA extraction and quantification

Bloodstains were collected in FTA cards, under informed consent, from unrelated males of three Nigerian groups: Hausa (n = 89), Yoruba (n = 135) and Igbo (n = 134). Samples were collected in different local governments and communities of Lagos State (the most cosmopolitan state of Nigeria). The ethnolinguistic affiliation of the individuals was traced back to three generations, with parents, grandparents and great-grandparents all belonging to the same ethnic group (ascertained by a questionnaire). DNA was extracted using the chelex method^13^. Quantification was performed by RT-PCR, using the Quantifiler Human DNA Quantification Kit (Applied Biosystems, Waltham, MA, USA). A total of 40 samples could not be typed for all three marker sets, due to low DNA quantity/quality. To ensure a good quality of the final data, incomplete profiles for Y-STRs, Y-SNPs or complete mtDNA control region were not included in the study.

### mtDNA typing

The entire control region (16024-576) of 324 samples was amplified as described by Simão et al*.*^14^, using one of the primers pair: L15900/H639, L15967/H20 and L16475/H639 (sequences are detailed in Supplementary Table S1)^15–17^.

The PCR products were purified using ExoSAP enzymes (Applied Biosystems) or ZYMO DNA Clean & Concentrator-5 (Zymo Research, Irvine, CA, USA).

Sequences were obtained using the BigDye v3.1 cycle Sequencing kit (Applied Biosystems), following the manufacturer’s guidelines, and the primers described in Supplementary Table S1.

The sequencing products were purified through illustra Sephadex DNA Grade columns (GE Healthcare, Chicago, IL, USA) or using the ZR DNA Sequencing Clean-up Kit (Zymo Research); and separated and detected on a 3500 Genetic Analyzer (Applied Biosystems).

Haplotypes were determined with the SeqScape v2.7 software (Applied Biosystems) or the Sequencher 5.4.6 software (Gene Codes, Ann Arbor, MI, USA), by comparison to the Revised Cambridge Reference Sequence (rCRS)^18^.

The conversion of mtDNA haplotype into sequences and the alignment were performed on Haplosearch^19^. In comparisons using published data, indels at positions 16030-16193, 16194-309, 310-315, 316-522, 525-573 and 574-576 were disregarded.

Haplogroups were assigned on EMPOP (https://empop.online), according to the Phylotree build 17, February 2016^20^. Data was submitted to the EMPOP database (https://empop.online/) for quality control and is available under the accession number EMP00856. Mitochondrial DNA sequences were deposited in GenBank: PopSet 2709404361 (https://www.ncbi.nlm.nih.gov/popset/?term=2709404361), accession numbers: PP578990-PP579313.

### Y chromosome typing

A total of 356 samples were genotyped for 27 Y-STRs using the Yfiler Plus PCR Amplification Kit (Applied Biosystems), according to the manufacturer’s protocol. PCR fragments were separated and detected on a 3500 Genetic Analyzer (Applied Biosystems). The GeneMapper ID software v4.0 (Applied Biosystems) was used for allele assignment.

A total of 351 samples were genotyped for 41 Y-SNPs (12f2a, 92R7, M2, M9, M13, M26, M30, M33, M35, M60, M62, M70, M75, M78, M81, M85, M96, M109, M112, M123, M150, M154, M168, M170, M172, M173, M182, M191, M201, M213, M293, P2, P25, SRY10831, Tat, U174, U209, U290, V6, V88 and YAP) (Supplementary Fig. S2).

In all samples, the Y Alu polymorphic insertion (YAP) was first genotyped in a single PCR as described in Gomes et al*.*^21^. Based on YAP results, additional SNPs were selected and genotyped through PCR and single-base extension sequencing using the SNaPshot Multiplex Kit (Applied Biosystems). The V88 was typed by Sanger sequencing, as described in González et al.^22^. The remaining 39 Y-SNPs were included in 5 multiplexes previously described by Brión et al.^23^ (Multiplexes 1 and 2), Gomes et al.^19^ (Multiplexes B and E2) and Rodrigues et al.^24^ (Multiplex E1).

In comparisons using published data, Y chromosome haplotypes were reduced to 17 Y-STRs, the common set of markers among the populations selected for comparisons.

### Data analyses

Haplotype (HD) and haplogroup (HgD) diversities were calculated using the formula implemented in the software Arlequin ver. 3.5.1.2^25^: $\widehat{H}= \frac{n}{n-1} \left(1-\sum_{i=1}^{k}{{p}_{i}}^{2}\right)$, where n is the sample size, k is the number of haplotypes/haplogroups and *p*_i_ is the frequency of the *i*-th haplotype/haplogroup. The same software was used to calculate the Mean Number of Pairwise Differences (MNPD) between all pairs of haplotypes in the sample, using the formula: $\widehat{\pi }= \frac{n}{n-1} \sum_{i=1}^{k}\sum_{j=1}^{k}{p}_{i}{p}_{j}{\widehat{d}}_{ij}$, where n is the sample size, k is the number of haplotypes, *p*_i_ is the frequency of the *i*th haplotype and ${\widehat{d}}_{ij}$ is an estimate of the number of mutations between haplotypes. Analyses of molecular variance (AMOVA) and genetic distances with corresponding non-differentiation probabilities were calculated using the software Arlequin ver. 3.5.1.2^25^. Genetic distances were based on the number of different alleles (*F*_ST_) for mtDNA, Y-STRs and Y-SNPs^26,27^; the sum of squared size differences (*R*_ST_) for Y-STRs^28^; and nucleotide differences (Nei’s average number of pairwise differences within and between populations) for mtDNA^29^. Pairwise *F*_ST_ genetic distance matrices were represented in two-dimensional plots using the multidimensional scaling (MDS) analysis included in the STATISTICA data analysis software system, ver.8.0 (TIBCO Software Inc., Palo Alto, CA, USA). The same software was used to perform Principal Component Analysis (PCA) based on Y-SNP haplogroup frequencies in populations. In MDS analysis, Nei’s distances were converted to percentage of variation by dividing the corrected net number by the average number of nucleotide differences between populations. Networks were designed applying reduced median and median-joining methods, as implemented in the Network v10.1.0.0 software (Fluxos Technology Ltd., Colchester, UK). For the Y-chromosomal STRs, weights were assigned inversely proportional to their variance.

### Ethical approval

This study was approved by the Health Research Ethics Committee from the Lagos University Teaching Hospital, assigned number: ADM/DCST/HREC/APP/540. The ethical principles of Helsinki Declaration of the World Medical Association were followed, and informed consent was obtained from all participants.

## Results

### Genetic diversity in Nigerian populations

The mtDNA and Y-STR haplotypes and corresponding haplogroups obtained in this study are listed in Supplementary Table S2. A total of 94 different mtDNA haplogroups were detected, 36 of which were observed only once. For the Y-Chr, 17 different haplogroups were detected, 7 of them observed in only one sample.

**Figure 1:**
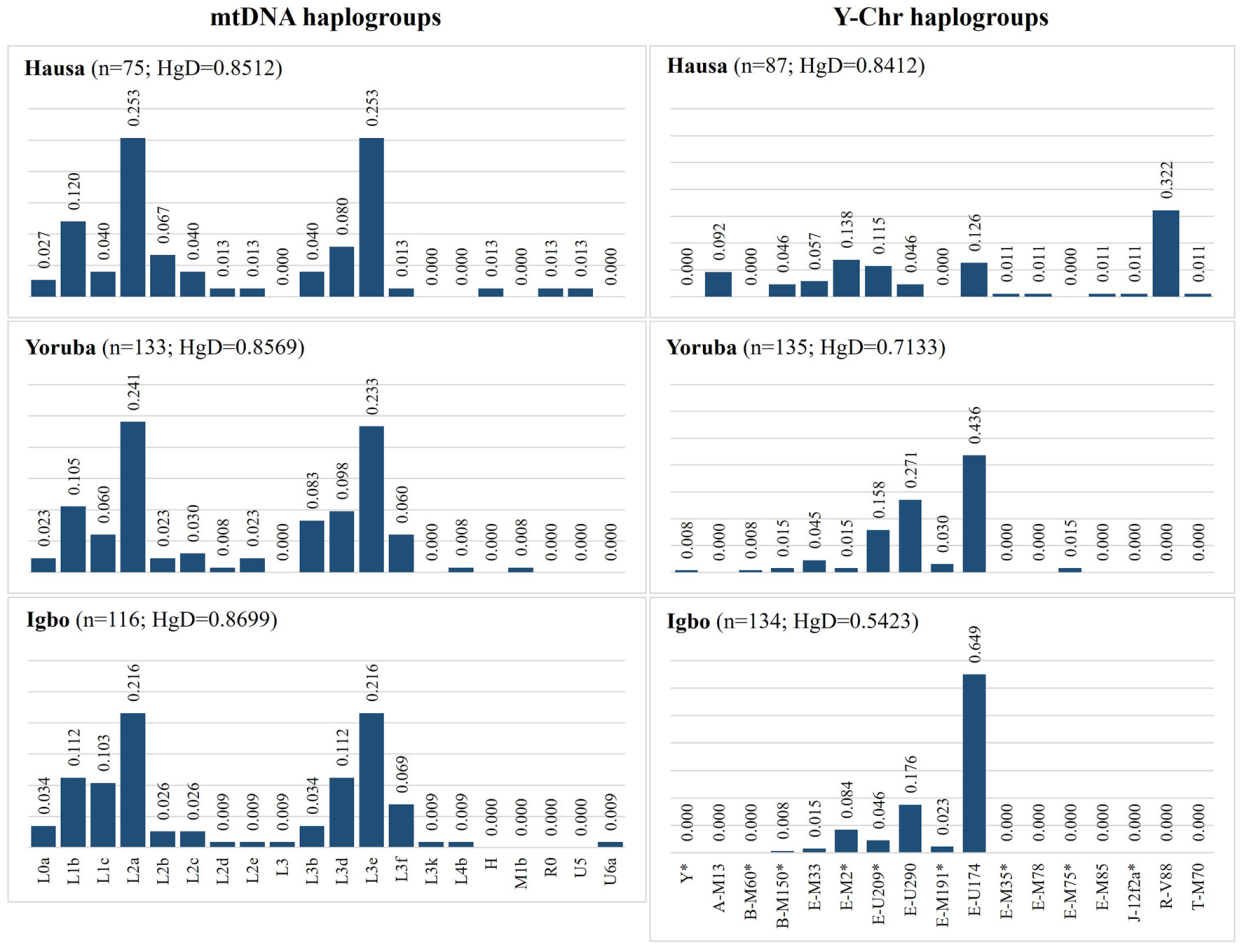
Frequency distributions of mtDNA and Y-SNP haplogroups in the Hausa, Yoruba and Igbo populations from Nigeria, and corresponding values of diversity (HgD). For mtDNA, the 20 haplogroups in the figure represent 94 different sub-haplogroups detected in our samples.

The frequency distributions of the main mtDNA and Y-Chr haplogroups in the three ethnic groups are represented in Fig. 1. The three Nigerian groups showed a similar distribution of mtDNA haplogroups. Although the number of mtDNA haplogroups was higher in the Hausa than in the Yoruba and Igbo samples, it presented a slightly lower diversity (Fig. 1) due to a less even distribution of the most frequent haplogroups. A much more heterogeneous pattern was observed in the frequency distributions of Y haplogroups among the three groups. The Hausa showed the highest diversity of Y-Chr haplogroups, with the most frequent lineage R-V88 not being present in the Yoruba and Igbo samples. The Igbo showed a low Y-Chr haplogroup diversity (Fig. 1), due to a high prevalence of the E-U174 lineage and a low number of different haplogroups.

Haplotype diversities for the entire mtDNA control region and for the 27 Y-STRs were above 99% in the three population groups (Table 1). For mtDNA, one haplotype was shared by two individuals in Hausa, 5 in Yoruba and 7 in Igbo. For the Y chromosome, no shared haplotypes were detected in Hausa, while one haplotype occurred twice in Yoruba and another haplotype was detected in three Igbo samples. To explore haplotype sharing within and between the three ethnolinguistic groups, networks were constructed for the major haplogroups (haplotypes inside mtDNA haplogroups L2a and L3e; and Y-Chr haplogroup E-U174). For mtDNA, haplotypes are spread among the three groups of Nigeria, with few haplotypes being shared among populations. No ethnic specificity was detected, even if considering close haplotypes (Supplementary Figs. S3, S4). For the Y-STRs, extremely reticulated networks were obtained (given the high mutation rates and recurrence of the STRs) that were difficult to visualize. Aiming to achieve a better resolution of the networks, further analyses were performed by retaining only the most stable loci—considering only loci with variances up to 0.5 (13 loci) and 0.3 (9 loci) as cutoff values (Supplementary Fig. S5). The reduction to 9 Y-STRs allowed a better resolution of the network, although with high haplotype sharing, intermingling in the three ethnic groups, not being informative of any kind of groups’ interactions or population substructure.

**Table 1:** Haplotype diversities (HD) and mean number of pairwise differences (MNPD) observed for the entire mtDNA control region and the 27 Y-STR haplotypes in Hausa, Yoruba and Igbo population groups.

	n	No. of different haplotypes	HD	s.e	MNPD	s.e
mtDNA Control region						
Hausa	75	74	0.9996	0.0023	16.4937	7.4276
Yoruba	133	128	0.9994	0.001	15.7629	7.0776
Igbo	116	109	0.9990	0.0013	16.9845	7.6092
27 Y-STR haplotypes						
Hausa	87	87	1.000	0.0017	18.3948	8.2352
Yoruba	135	133	0.9998	0.0009	15.3822	6.9135
Igbo	134	131	0.9997	0.0010	14.4446	6.5111

For both mtDNA and Y-STRs, the Hausa presented the highest values of haplotype diversity, followed by Yoruba and Igbo (Table 1). The same trend was observed for the MNPD between Y-STR haplotypes. Nonetheless, the MNPD between mtDNA sequences was higher in the Igbo than in the other groups, showing the same trend observed for the haplogroup diversities (Fig. 1).

The diversity values obtained for the three Nigerian groups were further compared with those for other African populations (Supplementary Tables S3, S4). It should be noted that many studies included samples from the general population of the country without dividing by ethnic groups, which could contribute to a greater diversity found with respect to the works in which the different groups are analyzed separately.

The highest overall values of mtDNA haplotype diversity were found in West, North and East regions of Africa, except for nomadic or semi-nomadic groups, namely the Tuareg and the Fulani, which present the lowest values of diversity (Supplementary Table S3), as previously reported^30^. In contrast, MNPD values are higher in populations from East and Southeast Africa, with populations from the West region, and in particular the Nigerian groups, showing intermediate MNPD values. This contrast between the haplotypic and nucleotide diversities in populations of southeastern Africa was also reported in other studies, being justified by a Khoisan substrate that would have persisted at the extreme of the Bantu expansion^31^. For the East African region, the high value of both HD and MNPD can be explained by the confluence of well-differentiated ethnolinguistic groups^32^.

Based on 17 Y-STRs, high values of HD were found in all populations (Supplementary Table S4). Contrasting with the similarity of haplotype diversity values, the MNPD have a high variation among populations. The high MNPD found in Hausa is comparable in scale to the values found in populations from East Africa. Because MNPD based on STR data do not account for the number of mutational steps underlying haplotype differences, these values are compatible with the admixture of male lineages belonging to well-differentiated groups, rather than the accumulation of diversity over time. The MNPD values found in the Yoruba are close to those of other populations in the Central-West region, while the Igbo has one of the lowest MNPD reported for African populations.

### Differentiation analysis among Nigerian populations

Analysis of molecular variance (AMOVA) was performed for the total mtDNA control region, with the three populations included in a single group. Most of the genetic variation was due to differences inside rather than among populations (Table 2). No statistically significant pairwise *F*_ST_ values were found among Hausa, Yoruba, and Igbo groups (Table 3). The same results were obtained when AMOVA and *F*_ST_ genetic distances were further calculated using mtDNA haplogroup frequencies.

**Table 2:** Results from the Analysis of Molecular Variance (AMOVA) based on the mtDNA entire control region haplotypes, and corresponding haplogroups, and for the 27 Y-STR haplotypes and Y-SNP haplogroups.

	Variation among populations (%)	Variation within populations (%)	*p* value*
mtDNA haplotype-based *F*_ST_^1^	− 0.21	100.21	0.7908
mtDNA haplogroup-based *F*_ST_^2^	− 0.12	100.12	0.7683
Y-Chr STR-based *F*_ST_^3^	4.83	95.17	< 5E^−6^
Y-Chr STR-based *R*_ST_^4^	8.12	91.88	< 5E^−6^
Y-Chr SNP-based *F*_ST_^5^	13.37	86.63	< 5E^−6^

^1^No. of different alleles (*F*_ST_), based on mtDNA haplotypes; ^2^Conventional F-Statistics from mtDNA haplogroup frequencies; ^3^No. of different alleles (*F*_ST_), based on Y-STR haplotypes; ^4^Sum of squared size difference (*R*_ST_), based on Y-STR haplotypes; ^5^Conventional F-Statistics from Y-SNP haplogroup frequencies; *p value obtained after 50,175 permutations.

**Table 3:** Results from pairwise genetic distance analyses based on the mtDNA entire control region haplotypes, and corresponding haplogroups, and for the 27 Y-STR haplotypes and Y-SNP haplogroups.

	Hausa versus Yoruba	Hausa versus Igbo	Yoruba versus Igbo
mtDNA haplotype-based *F*_ST_^1^	− 0.0036	− 0.0004	− 0.0023
Non-differentiation *p* value*	0.846	0.461	0.768
mtDNA haplogroup-based *F*_ST_^2^	− 0.0005	− 0.0007	− 0.0018
Non-differentiation *p* value*	0.525	0.589	0.848
Y-Chr STR-based* F*_ST_^3^	0.0634	0.0826	0.0120
Non-differentiation *p* value*	< 5E^−6^	< 5E^−6^	2E^−5^
Y-Chr STR-based *R*_ST_^4^	0.0957	0.1187	0.0411
Non-differentiation *p* value*	< 5E^−6^	< 5E^−6^	< 5E^−6^
Y-Chr SNP-based *F*_ST_^5^	0.1473	0.2257	0.0456
Non-differentiation *p* value*	< 5E^−6^	< 5E^−6^	4.2E^−4^

^1^No. of different alleles (*F*_ST_), based on mtDNA haplotypes; ^2^Conventional F-Statistics from mtDNA haplogroup frequencies; ^3^No. of different alleles (*F*_ST_), based on Y-STR haplotypes; ^4^Sum of squared size difference (*R*_ST_), based on Y-STR haplotypes; ^5^Conventional F-Statistics from Y-SNP haplogroup frequencies; *p value obtained after 50,175 permutations.

For the 27 Y-STR haplotypes, AMOVA and pairwise genetic distances were performed based on *F*_ST_ and *R*_ST_ genetic distances. In both tests, AMOVA showed statistically significant differences among the three groups (Table 2). Statistically significant differences were also found in all pairwise comparisons between Hausa, Yoruba and Igbo (Table 3).

### Differentiation analysis among populations from Africa

Genetic distances and corresponding non-differentiation *p* values were calculated between populations from Africa (listed in Supplementary Tables S5–S7)^10–12,21,22,30,33–57^. For mtDNA, similar results were obtained in population comparisons based *F*_ST_ and Nei’s genetic distances (Supplementary Tables S5, S6). In both cases, MDS representations show a high dispersion of the Fulani, Tuareg and Daza nomadic groups (Supplementary Fig. S6). Together with the low diversities observed in these populations (Supplementary Table S3), this result can be explained by genetic drift due to low effective population sizes. A central cluster of populations with *F*_ST_s ≤ 0.01 and non-significant *p* values when compared to the populations from Nigeria is observed, including Togo, Ghana and Ivory Coast populations, independently from the ethnolinguistic groups. The remaining populations from the West region, and those from Central-West, are scattered on the MDS around the central cluster (Supplementary Fig. S6), and well separated from the populations in other regions of Africa.

For the Y-Chr, apart from the previously reported differences among the three Nigerian groups, significant *F*_ST_ genetic distances were also observed in the comparison with other African populations (Supplementary Table S7). As can be seen in the MDS plot (Supplementary Fig. S7), the distribution of populations better correlates with ethnolinguistic affiliation than the observed for the mtDNA. In the MDS, the two Niger-Congo groups, Yoruba and Igbo, cluster with populations with the same ethnolinguistic affiliation, and the Hausa stand closer to other Afro-Asiatic populations.

### Principal Component Analysis of Y chromosome haplogroups

**Figure 2:**
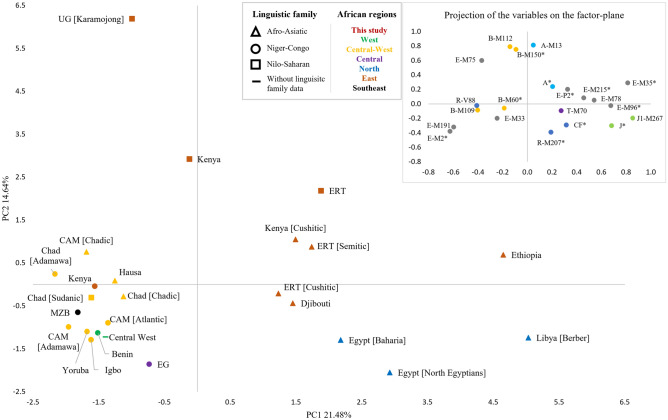
Scatterplot of the first (PC1) and second (PC2) principal components. Principal Component Analysis (PCA) was performed using haplogroup frequencies (as variables) in the three Nigerian samples and in other African populations (cases). CAM = Cameroon; EG = Equatorial Guinea; ERT = Eritrea; MZB = Mozambique; UG = Uganda.

A Principal Component Analysis (PCA) was performed to infer the most likely origin of the main Y-Chr haplogroups that are contributing to population differentiation. In this analysis, we used the frequency of 22 haplogroups obtained after retaining the maximum number of Y-SNPs in common among selected populations from Africa^21,22,43,44,48,49,53,58^ (Supplementary Table S8). In the PCA (Fig. 2), Igbo and Yoruba are located close to other Niger-Congo populations, which separates from most Afro-Asiatic populations in PC1, and Nilo-Saharans in PC2. The separation observed along PC2 can also be explained by geography and not by language, since the Nilo-Saharan populations are from East Africa, while the Chadic population is part of a central-western cluster. There are, however, exceptions to any of these patterns. On one hand, there is a Kenyan Niger-Congo group that appears to support linguistic separation. On the other hand, despite having different linguistic affiliations, the Chadic (Afro-Asiatic) and Sudanic (Nilo-Saharan) groups from Central-West stand close to the surrounding Niger-Congo populations. To assess the weight of each of these factors in the observed variation, AMOVAs were performed grouping populations based on geography or linguistics (Supplementary Table S9). For the two population groupings, a high variation within the groups was obtained, similar or higher than that found between them, showing that none of these criteria alone is sufficient to explain the existing variation. However, when the populations are grouped based on linguistics, we see less variation between populations within groups than when based on geography.

The main haplogroups contributing to the separation of Hausa from Yoruba and Igbo are (1) A-M13 and R-V88, only present in Hausa; and (2) E-M2 sub-lineages [E-M2* (xM191) and E-M191] that are prevalent in Yoruba and Igbo and less frequent in Hausa (Fig. 2). The haplogroup A-M13 has the highest frequency in Nilo-Saharan populations from East Africa^21,53^, but was detected in other Chadic populations with frequencies similar to Hausa^48^. The haplogroup R-V88, which is the most frequent in Hausa, has been associated to the dispersion of the Chadic languages and described at high frequencies in the region of Chad, north Nigeria, Cameroon and Niger^59,60^. The haplogroups E-M191 and E-M2* (xM191) are contributing to separate the West populations from the remaining, with Yoruba and Igbo having more than 90% frequency of these haplogroups. These are the most frequent lineages in sub-Saharan Africa, being absent or underrepresented in most populations from the North and East regions, outside the Niger-Congo family. Haplogroup B-M150* (xM109) also contributed to the separation of the three groups. Although present with low frequency, this haplogroup is more frequent in the Hausa than in the other two groups. It was not found in other Chadic groups, being frequent in Nilo-Saharan populations from East Africa^21,53^.

## Discussion and conclusions

The results obtained with the analysis of mtDNA and Y-Chr markers in the three major populations from Nigeria—Hausa, Yoruba and Igbo—and its comparison with other African populations allowed to deepen the knowledge on the interactions between ethnic groups in West Africa. Different scenarios regarding interactions mediated by women or men were observed, when contrasting the information provided by the two types of markers. Considering that the populations studied have traditionally been patrilocal and that polygyny is common to most Nigerian societies, we would expect Y-Chr genetic differentiation to be high between populations and low within populations, compared to mtDNA^61^. In fact, a higher Y-Chr than mtDNA differentiation among the studied populations was found, which supports a greater movement of the females^62^. Nonetheless, the expected decrease in Y-Chr diversity within populations due to polygyny was not observed in our samples.

### Female mediated genetic patterns

Similarities in the maternal lineage composition were found among the Hausa, Yoruba, and Igbo populations where the majority of mtDNA haplogroups were characteristic to sub-Saharan Africa. The Hausa group presented a slight difference with the two Niger-Congo samples, including few lineages, such as H and U5, which are more frequent in the Northern region of Africa (Fig. 1)^63,64^, and R0 lineage that is more frequent in the North and East regions^64,65^. The presence of these lineages in the Hausa group is likely the result of intense interactions with Islamic populations, during the trans-Saharan trade. The occurrence of these lineages is, however, residual, not being enough to demonstrate significant differences with the other two studied groups of Niger-Congo origin. The homogeneity observed between the three ethnic groups of Nigeria, as well as the high diversities found, are compatible with a continuous gene flow mediated by women. In agreement with the reported for other Nigerian groups from The Cross River region^9^, our results show that the gene flow occurred regardless of linguistic affiliations. Matrimonial practices may be behind this genetic homogeneity among Nigerian groups. Patrilocality, where newly married couples reside with or near the husband’s family, is a very common practice in several African populations, leading to a continuous movement of women among different ethnolinguistic groups. When expanding the analyses to other African populations, it was possible to see that this female-mediated gene flow extends to nearby populations from the West region, although influenced by the geographic distance. Our results allowed discerning significant differences between West and Central-West African populations, and a local homogenization of the female component, with more intense interactions between populations along the Gold Coast and Gulf of Benin.

### Male mediated genetic patterns

In opposition to the mtDNA, the Y-Chr revealed significant genetic distances among the three studied groups as well as differences in their diversity levels. The Hausa ethnic group was the most diverse considering both haplotype and haplogroup data. This diversity is characterized by the presence of typical haplogroups from East, North, and Central Africa, showing a genetic contribution to this population at the continental level. The Hausa not only presented the typical sub-Saharan African subclades inside haplogroups E-U209 and E-M191 (Fig. 1), but also a particular diversity of lineages from across Africa. Namely, the Hausa harbors: (1) lineages that are frequent in Nilotes from Sudan and Ethiopia in the East region (A-M13)^59^; (2) lineages that are more frequent in North and East African populations (E-M78)^66^; (3) a Middle Eastern haplogroup that is found in high frequencies in the North region (T-M70)^67^; (4) and a Proto-Chadic lineage (R-V88), with significant frequencies in the Central Sahel region of Africa and in Equatorial Guinea^22,60^. It is also worth highlighting the presence of a relatively high proportion of E-M2 lineages without the M191 or U209 mutations, which may belong to E-M2 subclades present in North Africa^59^. These results can be explained by ancient trade routes explored by men and by the natural connectors Sahel Corridor and Chad Basin. The presence of Chadic and Nilotic lineages found in the populations must have entered West Africa in more ancient times before the desertification of the Sahel as indicated in other studies^59^. Despite the diverse influences, the current Hausa group remained relatively differentiated from other neighboring groups from other ethnicities, showing a restricted gene flow at a microgeographic level.

A different pattern is observed in the other two groups from Nigeria. Although significant differences could be detected in all pairwise comparisons of the three groups, they were larger when involving the Hausa, which harbors many lineages that are not present in Yoruba and Igbo. On the other hand, the differentiation between Yoruba and Igbo is mainly due to differences in the frequency of the main haplogroups that are shared by both populations. Most lineages found in Yoruba and Igbo were from haplogroups inside E-M2 (mostly carrying M191 or U209 mutated alleles), lineages that are widely distributed in Niger-Congo populations in sub-Saharan Africa^59,68^. The Yoruba group has diversity levels that are typical of populations from Central-West Africa^47,48^, not showing signs of genetic drift that could evidence recent population bottlenecks. In turn, the Igbo shows a lower haplogroup diversity than the Yoruba, due to less even distribution of the frequencies. Based on historical records, a loss of diversity of Igbo male lineages could have happened during European colonization. The Igbo group, which had a small population contingent and was established near the ports of arrival, suffered from a massive loss of men, who were used for forced labor^2^. The involvement of Igbo people in the Biafran Civil War could also explain a decrease in haplogroup diversity. However, the high diversity at the Y-STR haplotype level is not compatible with such a recent drift effect. In fact, the high haplotype diversity inside haplogroup E sub-lineages is compatible with (1) an ancient drift event marked by the loss of haplogroup diversity and subsequent rapid expansion of the population, and/or (2) could be a reflection of the Y-SNPs selected for analysis in the present study. Given that most haplotypes found in Igbo were assigned to E-U174, a network analysis was performed (Supplementary Fig. S8) for this haplogroup. A high variation of haplotypes was observed, together with low haplotype sharing, pointing to the absence of important genetic drift events. In this manner, it can be assumed that the typing of more specific/downstream markers within this branch would allow distinguishing other sub-lineages. Therefore, both mentioned scenarios are compatible with the results. Despite the separation of Yoruba and Igbo, these populations share similar lineages, as expected due to their close origin and because they share the same language family.

On the other hand, the sharing of some haplogroups between the Yoruba and Hausa indicates some degree of gene flow between them. This result is somehow expected considering that adherence to Islam, the main religion of the Hausa, is not an isolated practice among the Yoruba. Such results point to a degree of communication between the Hausa and Yoruba and between Yoruba and Igbo that does not naturally occur between Hausa and Igbo in the male component. These differences in the Nigerian groups also indicate that the stratification of this component follows an ethnic pattern and not a geographical organization, in opposition to what was observed for the female component. In fact, when expanding the analyses to other African populations, and in accordance with the observed by Wood et al.^8^, the paternal genetic pattern of variation better correlates with ethnolinguistic affiliation. Nevertheless, linguistic alone cannot explain a high proportion of the existing variation. The two Niger-Congo groups, Yoruba and Igbo, are paternally genetically correlated with populations with the same ethnolinguistic affiliation, and the Hausa group is closer to other Afro-Asiatic populations.

### Final remarks

The present study aimed to fill existing gaps on the genetic composition of Nigerian populations. The genetic diversity of the three studied groups and their stratification is, in general, in agreement with the results of a recent study by Joshi et al.^69^. Based on whole genome data, these authors find a similar ancestral contribution between Yoruba and Igbo, and a different composition of the Hausa due to a shared ancestry with North African and European groups. Our study, due to the high geographic specificity of the uniparental (non-recombining) genomes provided interesting data and allowed a greater discrimination of the observed differences, complementary to whole genome data. With respect to mtDNA, the 3 groups have a closer ancestry than that found for biparental markers^69^. As for the Y chromosome, our results corroborate the genetic flow between the Hausa and North African populations^69^, also showing evidence of interaction with Nilo-Saharan groups. Moreover, we found no significant European influence in the Hausa, either female or male mediated, contrary to what was previously reported^69^.

By combining information from markers with exclusively maternal or paternal inheritance, it was also possible to demonstrate the impact of matrimonial practices, responsible for an intense female migration across linguistic borders, on the genetic composition of Hausa, Yoruba, and Igbo groups in Nigeria. By expanding our analyses to other African populations, it was possible to observe that the high genetic flow mediated by females is extensive to the Central-West populations. In contrast, the paternal lineages are much more sub-structured, which reinforces the maintenance of patrilocality as a regional practice. The high mobility of women for matrimonial purposes ends up being the mediator of a continuous gene flow, increasing the homogeneity in the maternal lineages and ethnic affinities between the Nigerian populations with surrounding countries. A higher correlation between genetics and geography indicates that language did not act as an important barrier to female-mediated gene flow. On the other hand, a higher differentiation is observed in the paternal lineages, which shows a better correlation with linguistic rather than geographical distances. However, neither of them alone is sufficient to explain the existing pattern of variation.

### Supplementary Information

Supplementary Information 1.

Supplementary Information 2.

## Data Availability

All data generated in this work is available in supplementary information.
