# Supplementary material for: Impact of patrilocality on contrasting patterns of paternal and maternal heritage in Central-West Africa
Source: Sci Rep. 2024 Jul 8;14:15653. doi: 10.1038/s41598-024-65428-z (PMC11231350; doi:10.1038/s41598-024-65428-z)
Supplement: Supplementary file 1 — Supplementary Information 1. [file 41598_2024_65428_MOESM1_ESM.pdf]

Supplementary Figures

Nguidi et al. (2024) Impact of patrilocality on contrasting patterns of paternal and maternal heritage in Central-West Africa. Scientific Reports

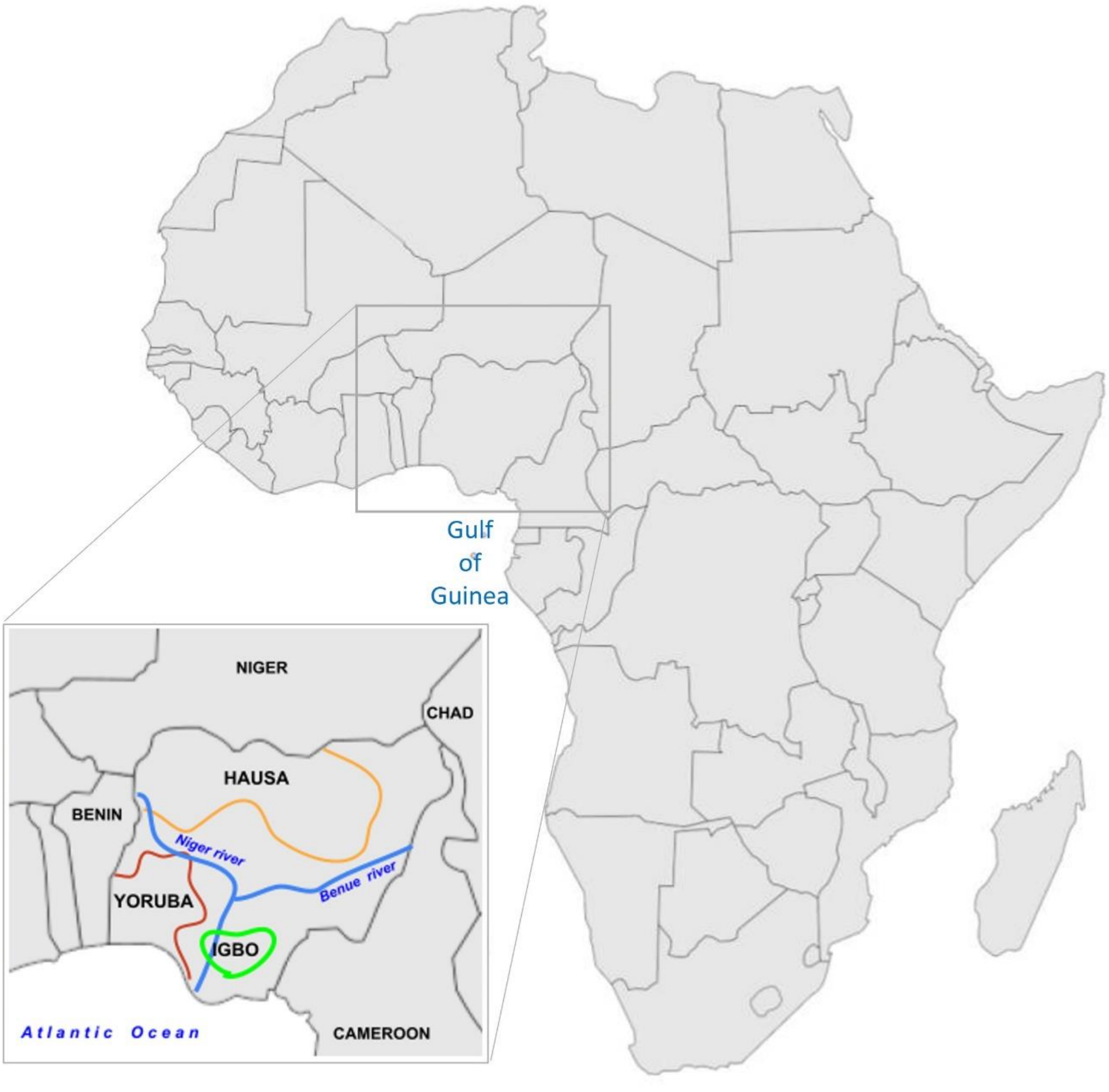

Supplementary Fig. S1 Map of Africa displaying Nigeria and neighboring countries in the West region of the continent. The current geographic distribution of Nigeria major ethnolinguistic groups is highlighted in different colors: Yoruba in red, Igbo in green, and Hausa in orange. The Niger and Benue Rivers are represented in blue. The background map in this figure was downloaded from “Collections of Pure PNG Images for Free Download” (https://www.vhv.rs/somore/africa).

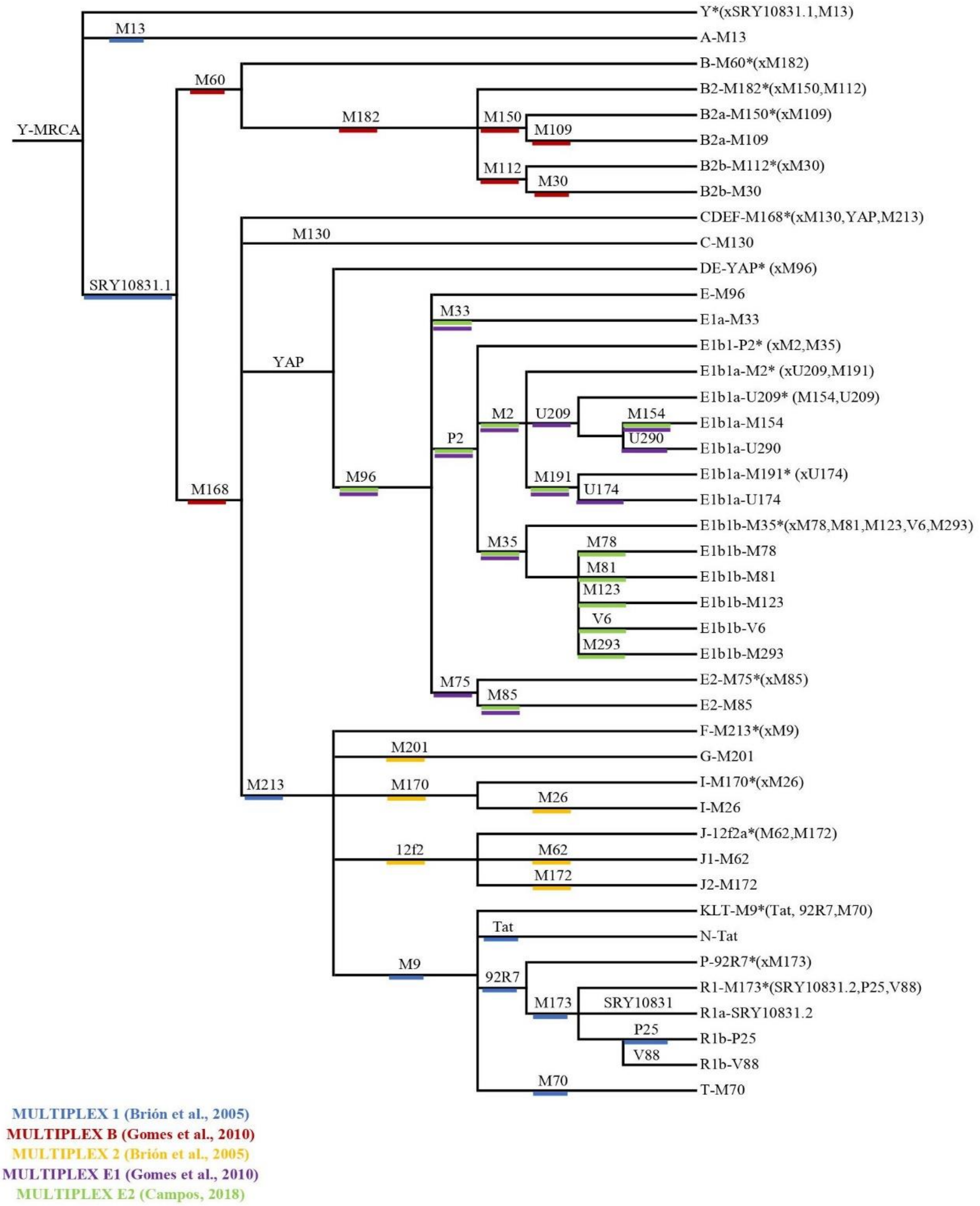

Supplementary Fig. S2 Phylogenetic tree of the Y-SNPs analyzed. The Multiplexes used in this study are differentiated by color.

References: Brion, M., Sobrino, B., Blanco-Verea, A., Lareu, M., Carracedo, A. Hierarchical analysis of 30 Y-chromosome SNPs in European populations. Int J Legal Med. 119, 10–15 (2005); Gomes, V., Sánchez-Diz, P., Amorim, A., Carracedo, A., Gusmão, L. Digging deeper into East African human Y chromosome lineages. Hum Genet. 127, 603–613 (2010); Rodrigues, P. et al. Tierra del Fuego: What is left from the precolonial male lineages? Genes. 13(10), 1712; 10.3390/genes13101712 (2022).

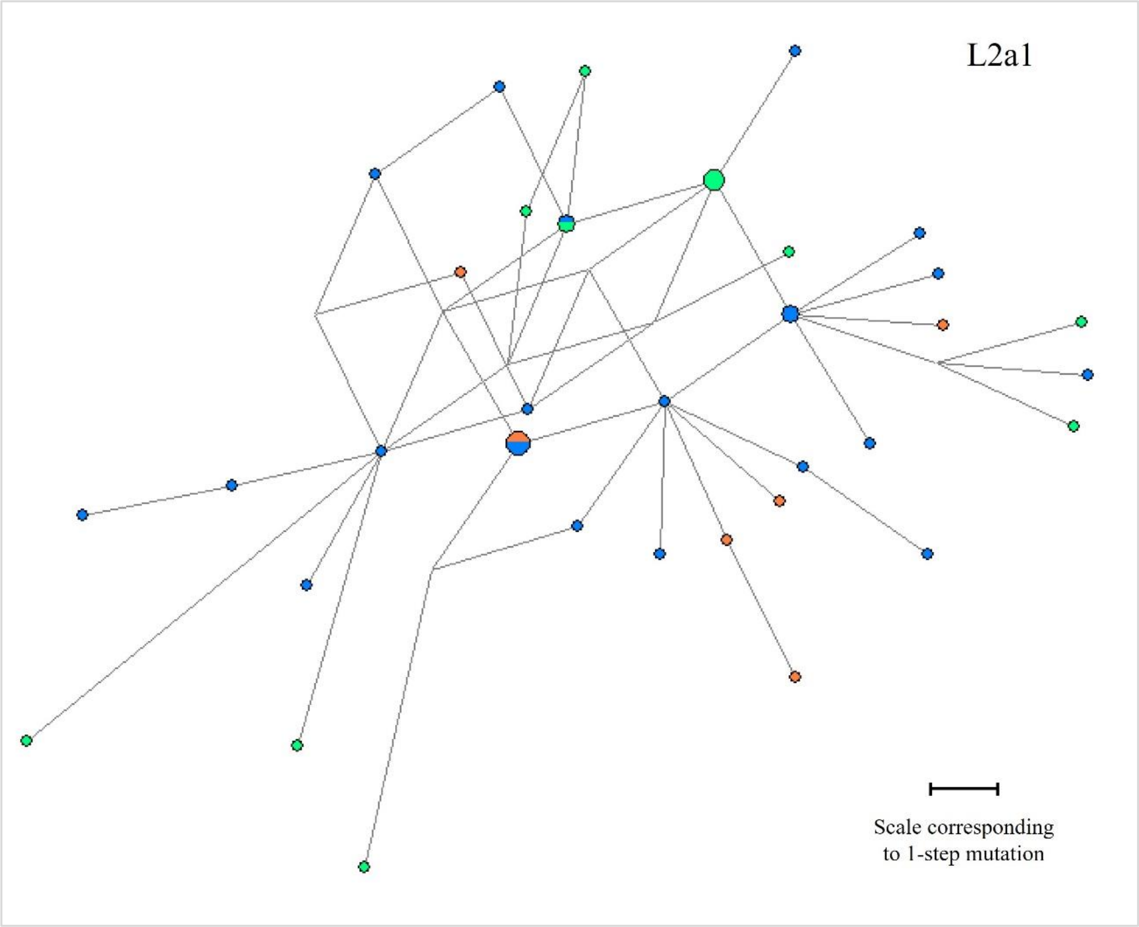

Supplementary Fig. S3 Network representation of control region haplotypes of the Hausa, Yoruba and Igbo samples assigned to the L2a1 haplogroup. The network was built using the Network v10.1.0.0 software, by applying the median-joining method. The size of the circles corresponds to the number of shared haplotypes. The number of mutated positions is proportional to the scale. The Nigerian populations are represented in different colors: Hausa (orange), Yoruba (blue) and Igbo (green).

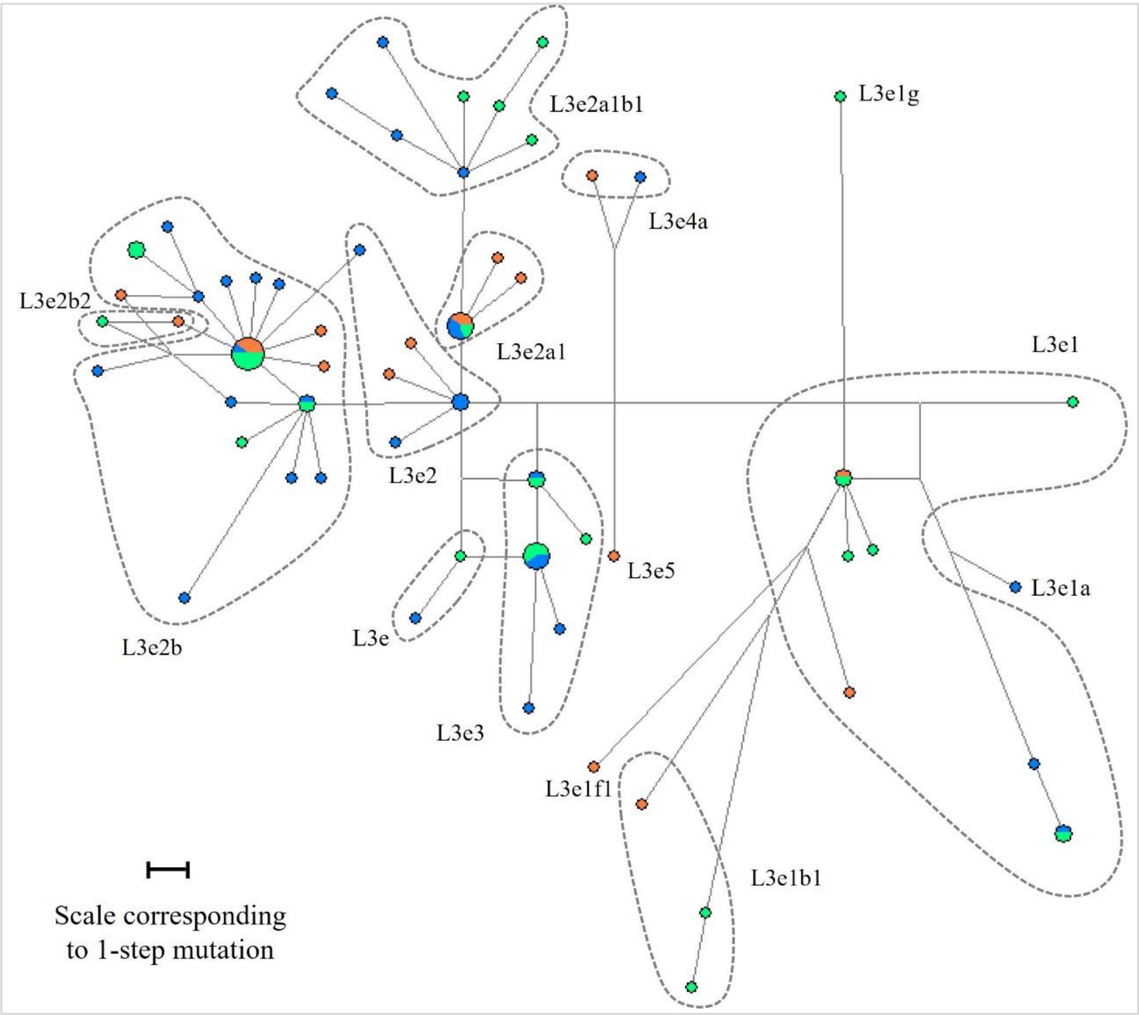

Supplementary Fig. S4 Network representation of control region haplotypes of the Hausa, Yoruba and Igbo samples assigned to the L3e haplogroup. The network was built using the Network v10.1.0.0 software, by applying the median-joining method. The size of the circles corresponds to the number of shared haplotypes. The number of mutated positions is proportional to the scale. The Nigerian populations are represented in different colors: Hausa (orange), Yoruba (blue) and Igbo (green).

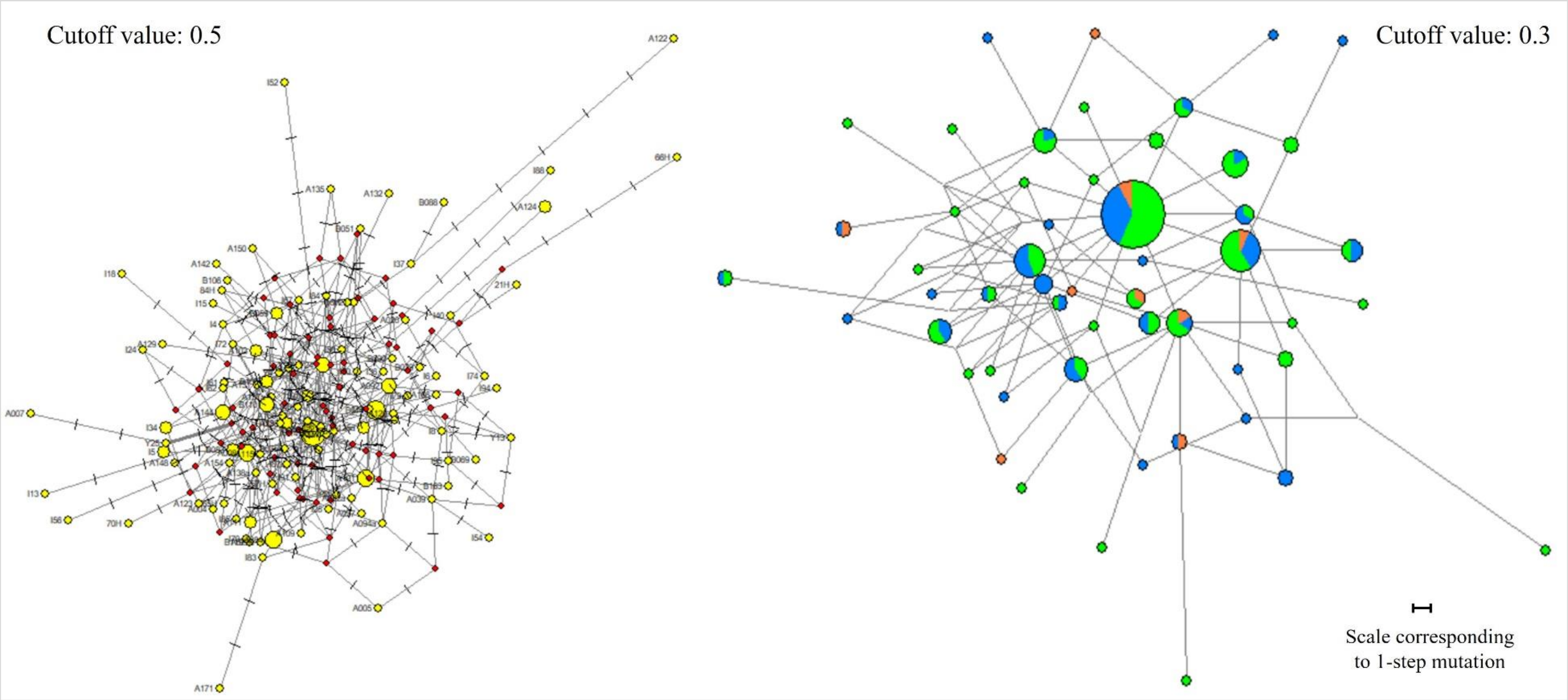

Supplementary Fig. S5 Network representation of Y-STR haplotypes in Hausa, Yoruba and Igbo, inside E-U174 haplogroup. The network was built using the Network v10.1.0.0 software and applying reduced median and median-joining methods sequentially. Differential weighting was applied to the Y-STRs, being inversely proportional to their variance. Aiming to resolve the networks, only the most stable loci were considered: (left side) 13 loci with variances up to 0.5 (DYS389I, DYS635, DYS389II, DYS460, GATAH4, DYS448, DYS391, DYS390, DYS438, DYS392, DYS437, DYS393 and DYS533); (right side) 9 loci with variances up to 0.3 (DYS389I, DYS389II, GATAH4, DYS391, DYS390, DYS438, DYS392, DYS437 and DYS533). The size of the circles corresponds to the number of shared haplotypes. The number of mutated positions is indicated along the main axis (left side) or proportional to the scale (right side). Red dots represent median vectors (left side). In the right side, the Nigerian populations are represented in different colors: Hausa (orange), Yoruba (blue) and Igbo (green).

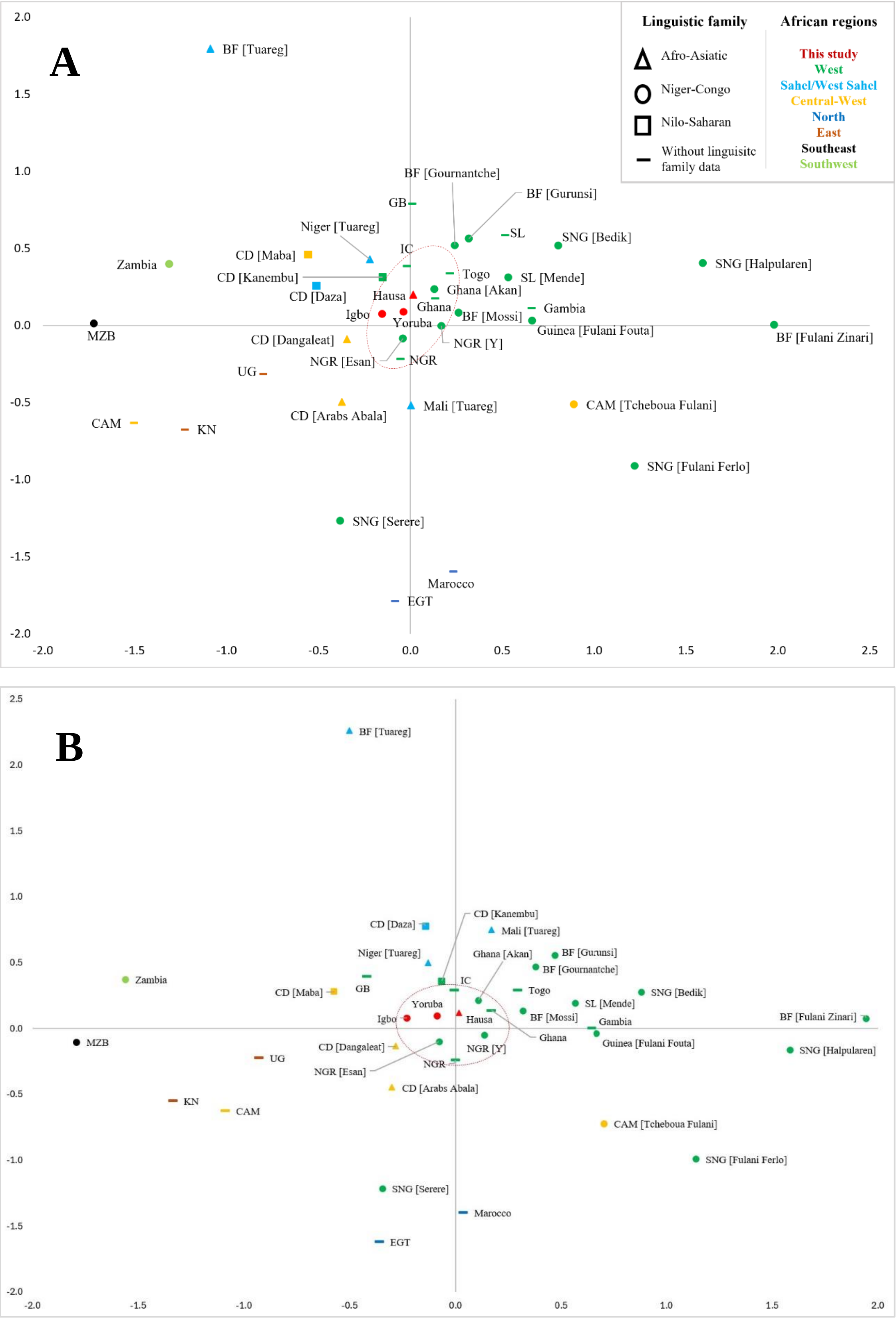

Supplementary Fig. S6 MDS plots of mtDNA genetic distances between the three ethnolinguistic groups of this study and populations from different regions of Africa. (A) MDS based on F_{ST} and (B) Nei's genetic distances. The central cluster (red dotted circle) include populations with distances ≤ 0.01 and non-significant *p*-values when compared to the populations from Nigeria.

BF = Burkina Faso; CAM = Cameroon; CD = Chad; EGT = Egypt; GB = Guinea-Bissau; IC = Ivory Coast; KN = Kenya; MZB = Mozambique; NGR = Nigeria; NGR [I] = Nigeria Igbo; NGR [Y] = Nigeria Yoruba; SL = Sierra Leone; SNG = Senegal; UG = Uganda.

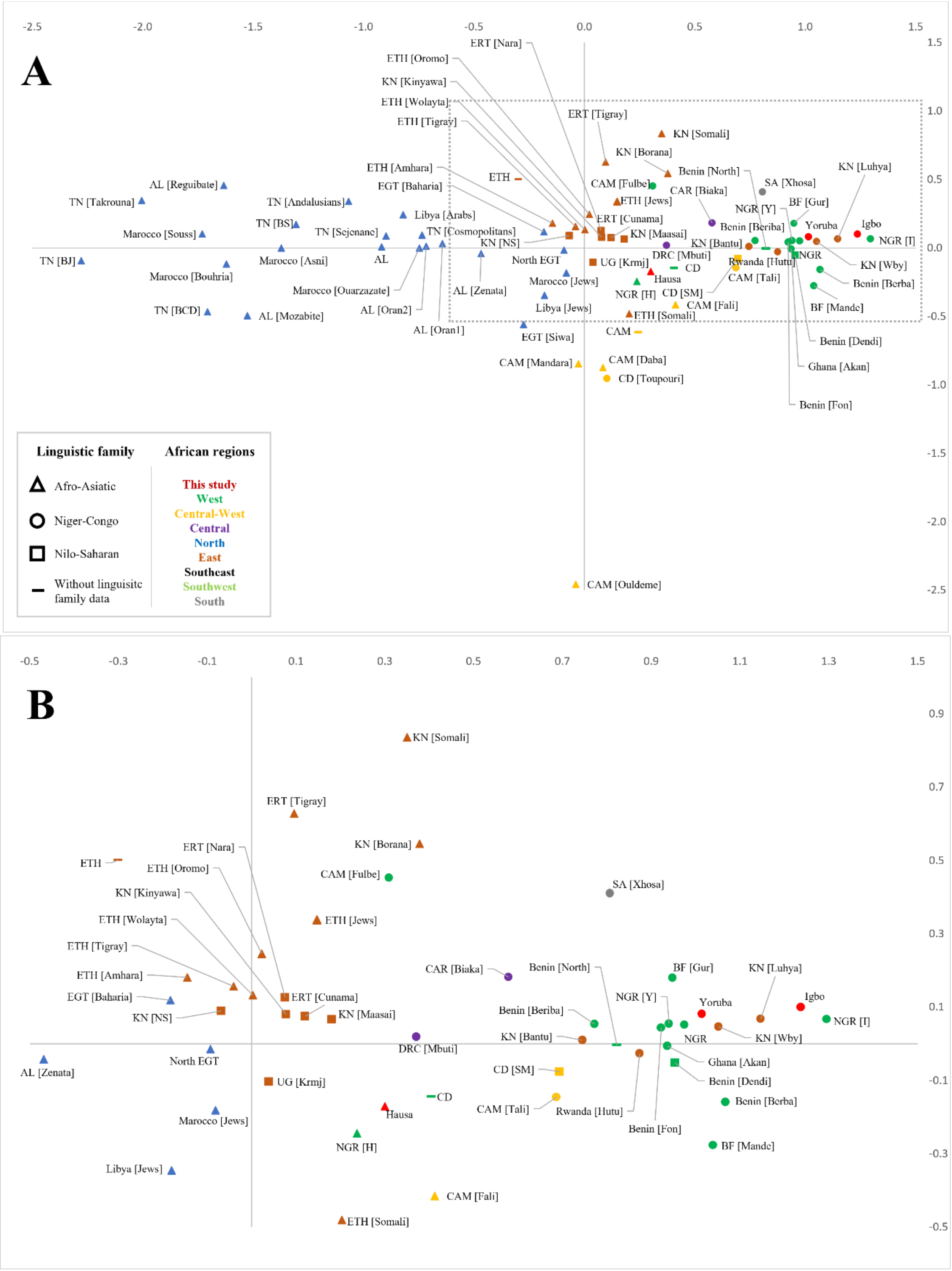

Supplementary Fig. S7 MDS plot of Y-STR genetic distances (F_{ST}) between the three ethnolinguistic groups from this study and populations from different regions of Africa. (A) All populations included; (B) detailed view of Nigerian and closer populations.

AL = Algeria; AN = Angola; BCD = Berbers Chenini Douiret; BF = Burkina Faso; BJ = Berbers Jradou; BS = Berbers Sened; CAM = Cameroon; CAR = Central African Republic; CD = Chad; DRC = Democratic Republic of Congo; EG = Equatorial Guinea; EGT = Egypt; ERT = Eritrea; ETH = Ethiopia; GB = Guinea-Bissau; IC = Ivory Coast; KN = Kenya; Kmrj = Karamojong; MZ = Mozambique; NGR = Nigeria; NGR [I] = Nigeria Igbo; NGR [Y] = Nigeria Yoruba; NS = Nilo-Saharans; SA = South Africa; SM = SaraMadjingay; SNG = Senegal; TN = Tunisia; UG = Uganda.

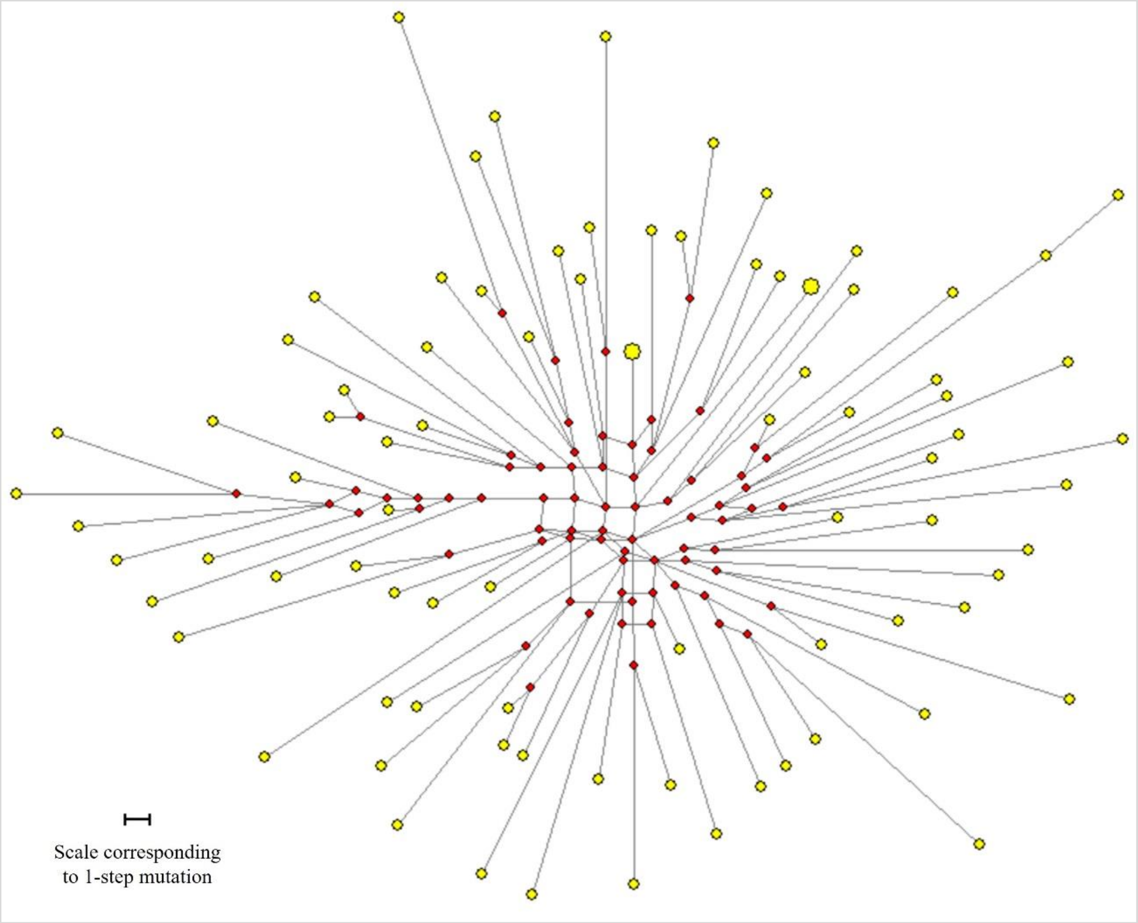

Supplementary Fig. S8 Network representation of Y-STR haplotype profiles of the Igbo samples assigned to the E-U174 haplogroup. Each yellow dot corresponds to one sample; red dots represent median vectors; and the mutated positions are indicated by lines along the main axes. The network was built using the Network v10.1.0.0 software (http://www.fluxusengineering.com) and applying the reduced median [Bandelt et al., 1995] and median-joining [Bandelt et al., 1999] methods sequentially. A total of 23 Y-STR loci were included (the two multi-loci markers DYS385 and DYF387S1 were not included). Differential weighting was applied, being inversely proportional to the STR variance [Qamar et al., 2002].

References: Bandelt, H. J. et al. Mitochondrial portraits of human populations using median networks." Genetics 141, 743-753 (1995); Bandelt, H. L. et al. Median-joining networks for inferring intraspecific phylogenies. Mol Bio Evol. 16, 37-48 (1999); Qamar, R, et al. Y-chromosomal DNA variation in Pakistan. Am J Hum Genet. 70, 1107-1124 (2002).
